# Sensing the cilium, digital capture of ciliary data for comparative genomics investigations

**DOI:** 10.1186/s13630-018-0057-0

**Published:** 2018-04-19

**Authors:** Karen R. Christie, Judith A. Blake

**Affiliations:** The Jackson Laboratory, 600 Main Street, Bar Harbor, ME 04609 USA

**Keywords:** Gene Ontology, Cilia, Ciliopathy, Ciliary-related disease, Flagella, Curation

## Abstract

**Background:**

Cilia are specialized, hair-like structures that project from the cell bodies of eukaryotic cells. With increased understanding of the distribution and functions of various types of cilia, interest in these organelles is accelerating. To effectively use this great expansion in knowledge, this information must be made digitally accessible and available for large-scale analytical and computational investigation. Capture and integration of knowledge about cilia into existing knowledge bases, thus providing the ability to improve comparative genomic data analysis, is the objective of this work.

**Methods:**

We focused on the capture of information about cilia as studied in the laboratory mouse, a primary model of human biology. The workflow developed establishes a standard for capture of comparative functional data relevant to human biology. We established the 310 closest mouse orthologs of the 302 human genes defined in the SYSCILIA Gold Standard set of ciliary genes. For the mouse genes, we identified biomedical literature for curation and used Gene Ontology (GO) curation paradigms to provide functional annotations from these publications.

**Results:**

Employing a methodology for comprehensive capture of experimental data about cilia genes in structured, digital form, we established a workflow for curation of experimental literature detailing molecular function and roles of cilia proteins starting with the mouse orthologs of the human SYSCILIA gene set. We worked closely with the GO Consortium ontology development editors and the SYSCILIA Consortium to improve the representation of ciliary biology within the GO. During the time frame of the ontology improvement project, we have fully curated 134 of these 310 mouse genes, resulting in an increase in the number of ciliary and other experimental annotations.

**Conclusions:**

We have improved the GO annotations available for mouse genes orthologous to the human genes in the SYSCILIA Consortium’s Gold Standard set. In addition, ciliary terminology in the GO itself was improved in collaboration with GO ontology developers and the SYSCILIA Consortium. These improvements to the GO terms for the functions and roles of ciliary proteins, along with the increase in annotations of the corresponding genes, enhance the representation of ciliary processes and localizations and improve access to these data during large-scale bioinformatic analyses.

**Electronic supplementary material:**

The online version of this article (10.1186/s13630-018-0057-0) contains supplementary material, which is available to authorized users.

## Background

Interest in cilia has increased dramatically over the last 10 years as it has become clear that ciliopathies are an underlying cause of numerous human diseases [1–5]. Notably, there has been a surge in the number of publications reporting advances in our understanding of ciliary biology. However, in this era of comparative genome analysis and bioinformatics, the data need to be available in a structured, digital format that is accessible to computational analysis in order to get the most out these recent insights into ciliary biology. To this end, we focused on functional annotation of ciliary genes for the laboratory mouse using the Gene Ontology (GO), and participated in a collaborative effort with the SYSCILIA Consortium and the Gene Ontology Consortium to expand and improve the formal, digital representation of ciliary biology within the GO [6–8].

Sperm and various types of epithelial cells have long been known to possess motile cilia. Then, since the 1970s, we learned that defects in ciliary motility are associated with numerous dysfunctional phenotypes that are now characterized as Primary Ciliary Dyskinesia or PCD, including chronic respiratory and sinus infections, male infertility, and reversal in the left/right organization of the body [9–11]. The motile cilia present on many types of multiciliated epithelial cells are essential for movement of fluids across tissues [12] and play important roles in the development and function of many organs including the brain [13], nasal and respiratory passages [14], and fallopian tubes [15].

While it has been known for over 100 years that many mammalian cell types possess a single non-motile cilum, also referred to as a primary cilium, these primary cilia were thought, until fairly recently, to be functionless evolutionary relics [1, 16]. It is now clear that primary cilia are sensory organelles critical to the regulation of many signaling pathways including Sonic hedgehog (*Shh*)—a major regulator of early developmental patterning [1–4, 16, 17]. There is evidence that motile cilia also possess sensory functions [18]. The motile primary cilia of the embryonic node play another key role in early development as they are required for the initial generation of left/right asymmetry in the embryo and thus are required for proper morphogenesis of asymmetric anatomical structures such as the heart and the digestive tract. Specialized cilia are part of structures required for detecting sensory input, such as the kinocilia at the center of stereocilia bundles in cochlear hair cells; the cilia of olfactory sensory neurons within olfactory epithelium; and the modified cilia of photoreceptor cells [16, 19, 20].

With this greater understanding of the diversity of both the structures and the roles of cilia [21], we are developing a greater understanding of the underlying common role of defects in ciliary function in developmental defects in right/left symmetry including *situs inversus* [22], brain development defects including hydrocephaly [13], congenital heart defects [23, 24], and craniofacial defects [25], and also with many diseases that manifest after birth or later in life, such as obesity [26–28], recurrent sinus and respiratory tract infections [9, 14, 22], hearing loss [29], vision loss [20, 30, 31], kidney disease [32–34], and infertility [9, 11, 35].

This increased understanding of the role of cilia in these various developmental defects and diseases, collectively referred to as ‘ciliopathies,’ drives a desire to better understand the genes involved in these human diseases. In cases of PCD, where a mutation in a specific gene leads to a loss of ciliary motility, but not loss of cilia entirely, it may be possible to characterize the ciliary defect in cells from affected human patients. However, it is not always possible to find human mutations relevant to the study of a particular gene, particularly if loss of that function results in embryonic lethality. Thus, mouse models can play a key role in developing our understanding of the role of cilia in development throughout the embryo [24] and in increasing our understanding of ciliary function in many human disease syndromes [36]. The ability to generate mice with gene knockouts specifically targeted for specific tissues or embryonic stages allows researchers to address questions that are not possible any other way. However, for the wealth of experimental data generated by these mouse models to be most useful, this information must be made digitally available for analysis of large-scale experiments such as enrichment analyses of gene lists resulting from high-throughput expression or phenotypic studies, or for comparative genome analyses.

To further the availability and utility of the experimental work on cilia, we initiated a project to comprehensively annotate experimentally characterized ciliary genes of the laboratory mouse using Gene Ontology (GO) terms to describe their molecular functions, biological roles, and cellular locations [6, 37]. Here, we describe our progress, including how our workflow provides a model for targeted annotation of genes from a model organism relevant to a specific human disease or health issue. Inspired by the publication of the SYSCILIA Consortium’s Gold Standard set of known human ciliary components, we initiated our work on the mouse equivalents of these known human ciliary components as targets for comprehensive GO annotation of mouse ciliary genes. We subsequently became aware of other excellent resources that have compiled sets of ciliary genes, such as Cildb [38], and these other genes may be part of other annotation projects in the future. As part of the curation process, we also updated the Gene Ontology as needed to best represent our understanding of ciliary biology [8]. Our experimental annotations to mouse genes gain additional value as they are propagated via phylogenetic methods to several related taxa including rat and human [39, 40]. This inference process improves the ability of researchers to identify a common ciliary role in the sets of genes identified in their research, regardless of whether they are looking at lists of human genes, or, by inference, of lists of genes from a model organism such as the mouse.

## Methods

### Conversion of SYSCILIA list of human ciliary genes to corresponding mouse genes

Ensembl gene IDs from the Excel file available from the SYSCILIA Consortium’s website [41, 42] were mapped to UniProt IDs using UniProt’s ID mapping service [43–45]). During this process, it was noticed (and reported to the SYSCILA Consortium for updating) that ENSG00000146038 (for *DCDC2*) was present twice, thus resulting in only 302 unique Ensembl IDs being converted to UniProt IDs for human protein sequences.

Mouse genes corresponding to the human genes on the SYSCILIA gold standard were identified using MouseMine [46, 47], which included homology data from both PANTHER [48] and HomoloGene [49] resources. Using the MouseMine “Genes ⇒ Homologs” template [50], we identified 297 mouse genes as 1:1 orthologs of their corresponding human genes, as well as 10 mouse genes with a 2:1 or 4:1 relationship between mouse genes and the corresponding human genes, producing a list of 307 mouse genes corresponding to 301 human genes. The remaining human gene (*SLC47A2*) did not result in any mouse orthologs using this MouseMine query. However, Hs *SLC47A2* is in PANTHER family PTHR11206, where the two mouse genes (*Slc47a1* and *Slc47a2*) were both placed in a duplication node more closely related to the other human gene *SLC47A1* than to human *SLC47A2* within Panther family PTHR11206 (PANTHER version 9.0). Although neither the Panther nor HomoloGene data indicated that either mouse gene is a homolog of human *SLC47A2* as both mouse genes are more closely related to the human *SLC47A1* gene than to *SLC47A2*, both mouse genes were added to the list of target genes to annotate to ensure we included the most closely related gene to human *SLC47A2*. After this initial mapping, we observed during the curation process that three mouse genes *Ttc30a1*, *Ttc30a2*, and *Ttc30b* were in the same PANTHER family (PTHR20931) as two human SYSCILIA Gold Standard genes (*TTC30A* and *TTC30B*), so *Ttc30a2* was added to the list of mouse genes based on these PANTHER family data which were not available at the time of the original mapping. Due to the structure of the PANTHER tree for PTHR20931, we have marked the orthology relationship between the two human and three mouse genes in this family as unclear. Combining the data from these sources, we focused our work on a list of 310 mouse genes that correspond to the genes on the SYSCILIA gold standard of human ciliary genes (see Table 1 and Additional file 1).

**Table 1:** Mouse equivalents of the SYSCILIA Gold Standard list of human ciliary genes

Homology data source	# genes with *m* human to *n* mouse relationship				Total
	1:1	1:2	1:4	Unclear	
MouseMine: both HomoloGene and PANTHER	264	4	1		269
MouseMine: HomoloGene only	27		3		30
MouseMine: PANTHER only	4	2			6
Manual examination of PANTHER family				5	5
Total	295	6	4	5	310

Starting with the 302 genes of the SYSCILIA Consortium’s Gold Standard list of human ciliary genes, we used a combination of HomoloGene and PANTHER data present in MouseMine to identify the corresponding mouse genes. For five genes, visual examination of PANTHER family trees was also used to determine which mouse genes were related to the human genes. These 310 mouse genes comprised our list of mouse genes to target for curation

### Acquisition of initial and final GO annotation sets

At the start of our project on 7/25/2013, all GO annotations for 304 mouse genes were obtained and downloaded via the MouseMine “Mouse features ⇒ Functions (GO terms)” template [51]. As the initial mapping did not include 6 of the mouse genes in the final set of 310, annotation data for these six mouse genes were recovered from the gene_association.mgi file Revision **10039** (dated *Fri Jul 12 02:45:51 2013 UTC*) downloaded from the Gene Ontology’s archive of MGI GAFs [52]. These data constitute the baseline prior to this curation project (see Additional file 2). The same MouseMine query [51] was used to download all annotations for all 310 genes on 12/24/2016 to generate the annotation set after targeted annotation of ciliary genes, and these were used for evaluation and testing of the impact of this work (see Additional file 3).

### Acquisition of data on associated references

References associated with these 310 genes were obtained and downloaded via the MouseMine “Mouse features ⇒ Publications” template [53] on 7/25/2013. All associated references were saved as a list. It was then determined which of these publications were already used for GO annotations. The List subtraction operation in MouseMine allowed generation of a list of publications associated with any of these genes and not yet curated for GO. The number of papers tagged for curation with GO and linked to each gene was determined via MGI’s internal curation database (see Additional file 1).

### Enrichment analyses

Enrichment analyses were performed using version 1.6.0 of the Visual annotation Display (VLAD) tool at Mouse Genome Informatics [54, 55]. For mouse annotation data, gene_association.mgi file Revision **10039** (dated *Fri Jul 12 02:45:51 2013*) was used for the July 2013 data set and Revision **37787** (dated *Fri Dec 23 03:46:12 2016)* was used for the December 2016 data set, both downloaded from the Gene Ontology’s archive of mouse GAFs that have passed GO quality control checks [52]. For the ontology data, gene_ontology.1_2.obo file Revision **1.1190** (dated *Wed Jul 24 17:54:23 2013 UTC*) was selected for the July 2013 data and Revision **1.1973** (dated *Fri Dec 23 19:16:48 2016 UTC*) was selected for the December 2016 data set, both downloaded from the archive of GO ontology files [56]. Only annotations with experimental evidence codes IDA, IGI, IMP, IPI, IEP, and EXP (the latter two of which are not present in the July 2013 GAF) were included for the enrichment analysis. See Additional file 4 for expansions of evidence code acronyms. See Additional file 5 for the tabular results of the analysis using the July 2013 data and Additional file 6 for the results of the analysis using the December 2016 data.

## Results

### Mouse orthologs of human genes in SYSCILIA Gold Standard set

Using a combination of PANTHER [48] and HomoloGene [49] orthology data, both available via MouseMine [47, 50], we identified 307 mouse genes that are an ortholog or member of a gene family of the 301 unique human genes on the SYSCILIA Gold Standard list. By manual examination of PANTHER families, we added an additional mouse gene for one of these 301 human genes, as well as two mouse genes most closely related to the remaining human gene where MouseMine did not contain any results, for a total of 310 mouse genes on our list to target for curation efforts (see Table 1).

### Initial ciliary curation status

**Fig. 1:**
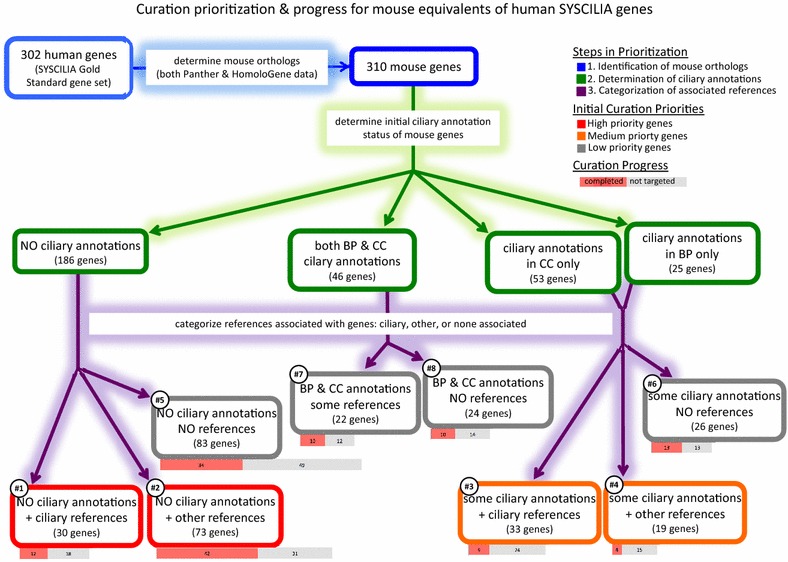
Curation prioritization and progress for mouse equivalents of human SYSCILIA genes. This flow chart diagrams our process of prioritizing the mouse equivalents of the human SYSCILIA Gold Standard list, starting with determining the mouse orthologs or most closely related gene(s), determining the state of existing ciliary annotations to the BP and CC terms used in July 2013 (as listed in Table 3), and determining the type of literature available (ciliary or other), if any. Genes lacking any ciliary annotations and with associated references comprised the high-priority category. Genes with annotations in only one aspect of GO (either BP or CC) and with associated references comprised the medium priority genes. Genes that were annotated with at least one ciliary GO term from both BP and from CC (see Table 4) were part of the low-priority category. The remainder of the low-priority category consisted of genes which were not associated with any references in July 2013, indicating that there were no publications available for curation of these genes at that time. Bars below each numbered bubble indicate the number of genes in that grouping that were “completely” curated and the number which were not targeted for curation as of December 2016

To determine initial annotation status of these genes with respect to ciliary terms and processes as of 7/25/2013, we evaluated all experimental GO annotations (excluding those with a NOT qualifier), a total of 3462 annotations for the 310 mouse cilia genes (see Table 2A). We identified 48 GO terms, 27 in biological process (BP) and 21 in cellular component (CC) specifically related to cilia or flagella (see Table 3), used in 349 experimental annotations of these genes. Of the 310 mouse genes, a small subset (46) were well annotated with ciliary terms from both the BP and CC aspects of GO. Another 78 genes were annotated with ciliary GO terms from either BP or CC, but not both. However, more than half (186) of the identified mouse genes had no experimental annotations to any ciliary term, and 91 of these genes had no experimental annotations whatsoever. Based on the existence of ciliary GO annotations in the BP and/or CC aspects, each gene was assigned to a “ciliary curation status” category (“Both BP and CC,” “BP only,” “CC only,” or “No ciliary annotations”) as a crude indication of possible priority need for further annotation to ciliary GO terms (see Table 4 and Fig. 1).

**Table 2:** Annotation counts from 2013 to 2016

Curation status	GO term category	Experimental	Unknown	Curator statement	Author statement	Sequence	Phylogenetic	Electronic	Total
A. Annotation counts in July 2013									
Completed genes (134)	Ciliary	153	–	0	5	79	0	41	278
	Related	225	–	0	3	92	0	16	336
	Other	316	87	0	18	328	0	598	1347
Not yet targeted genes (176)	Ciliary	196	–	3	0	74	1	18	292
	Related	705	–	0	6	168	12	22	913
	Other	1867	91	2	56	1192	49	934	4191
	Total	3462	178	5	88	1933	62	1629	7357
B. Annotation counts in December 2016									
Completed genes (134)	Ciliary	504	–	21	1	214	45	37	822
	Related	511	–	0	3	149	17	17	697
	Other	689	41	3	7	525	81	618	1964
Not yet targeted genes (176)	Ciliary	317	–	4	0	158	20	18	517
	Related	830	–	0	6	214	29	25	1104
	Other	2344	54	4	65	1712	216	905	5300
	Total	5195	95	32	82	2972	408	1620	10,404
C. Difference in annotation counts from 2013 to 2016									
Completed genes (134)	Ciliary	351	–	21	− 4	135	45	− 4	544
	Related	286	–	0	0	57	17	1	361
	Other	373	− 46	3	− 11	197	81	20	617
Not yet targeted genes (176)	Ciliary	121	–	1	0	84	19	0	225
	Related	125	–	0	0	46	17	3	191
	Other	477	− 37	2	9	520	167	− 29	1109
	Total	1733	− 83	27	− 6	1039	346	− 9	3047
D. Fold change in annotations from 2013 to 2016									
Completed genes (134)	Ciliary	3.29	–	DZ	0.20	2.71	DZ	0.90	2.96
	Related	2.27	–	DZ	1.00	1.62	DZ	1.06	2.07
	Other	2.18	0.47	DZ	0.39	1.60	DZ	1.03	1.46
Not yet targeted genes (176)	Ciliary	1.62	–	1.33	DZ	2.14	20.00	1.00	1.77
	Related	1.18	–	DZ	1.00	1.27	2.42	1.14	1.21
	Other	1.26	0.59	2.00	1.16	1.44	4.41	0.97	1.26
	Total	1.50	0.53	6.40	0.93	1.54	6.58	0.99	1.41

These tables show the number of annotations (excluding annotations with the NOT qualifier) by evidence type for the 310 mouse ciliary genes corresponding to the SYSCILIA Gold Standard set of human ciliary genes before our annotation project commenced in July 2013 (Panel A) and as of December 2016 (Panel B). The difference in the number of annotations from July 2013 to December 2016 is shown in Panel C and the fold difference in Panel D. “Completed genes” are those which have been annotated as fully as possible as of December 2016, while “genes not yet targeted” are those which have not yet been focused on specifically for comprehensive curation. However, some of these genes have received additional annotations during the project, generally due to being present in the same references as genes which were targeted for curation. GO annotations are categorized by type of evidence code. “Experimental” includes these evidence codes: IDA, IMP, IGI, IPI, and IEP. “Unknown” refers to annotations to the root node of each of the three aspects of GO using the ND evidence code indicating that nothing is known. As the three root terms are not part of either the ciliary or related term sets, a dash indicates that it is not possible to have unknown annotations in these categories. “Author statement” evidence includes both TAS and NAS codes. “Curator statement” refers to annotations using the evidence code IC. “Sequence” includes ISO, ISA, ISS, and ISM. “Phylogenetic” includes annotations made with the evidence code In IBA using the PAINT tool for phylogenetic annotation. “Electronic” refers to annotations made with the Inferred from IEA code, for example, annotations made on the basis of the presence of a specific InterPRO domain. For more information and expansions of the evidence code acronyms, see Additional file 4. The presence of DZ in a cell in part D indicates that there were zero annotations in 2013, making it impossible to calculate fold change

**Table 3:** Ciliary GO terms used in experimental annotations of mouse genes

#	GO aspect	GO ID	GO term name	Used in 2013	Used in 2016	Note
1	BP	GO:0001539	Ciliary or bacterial-type flagellar motility	Y	N	Term was too general for annotation of mouse genes; annotations were moved to a more specific term
2	BP	GO:0035083	Cilium axoneme assembly	Y	N	Merged into GO:0035082 “axoneme assembly”
3	BP	GO:0042384	Cilium morphogenesis	Y	N	Originally named “cilium assembly” before merge of GO:0060271 and GO:0042384 (IDs switched)
4	BP	GO:0035058	Non-motile primary cilium assembly	Y	N	Merged into GO:1905515 “non-motile cilium assembly”
5	CC	GO:0035085	Cilium axoneme	Y	N	Merged into GO:0005930 “axoneme”
6	CC	GO:0031512	Motile primary cilium	Y	N	Merged into GO:0031514 “motile cilium”
7	CC	GO:0031513	Non-motile primary cilium	Y	N	Merged into GO:0097730 “non-motile cilium”
8	CC	GO:0072372	Primary cilium	Y	N	Merged into GO:0005929 “cilium”
9	BP	GO:0070286	Axonemal dynein complex assembly	Y	Y	Modified by GOC/SYSCILIA project
10	BP	GO:0060404	Axonemal microtubule depolymerization	Y	Y	Modified by GOC/SYSCILIA project
11	BP	GO:0035082	Axoneme assembly	Y	Y	Modified by GOC/SYSCILIA project
12	BP	GO:0060830	Ciliary receptor clustering involved in smoothened signaling pathway	Y	Y	Modified by GOC/SYSCILIA project
13	BP	GO:0060271	Cilium assembly	Y	Y	Modified by GOC/SYSCILIA project; ID changed due to merge with GO:0042384 to remove “cilium morphogenesis” as a GO term
14	BP	GO:0003341	Cilium movement	Y	Y	
15	BP	GO:0044782	Cilium organization	Y	Y	Added by GOC/SYSCILIA project
16	BP	GO:0060285	Cilium-dependent cell motility (a)	Y	Y	Modified by GOC/SYSCILIA project
17	BP	GO:0003351	Epithelial cilium movement	Y	Y	
18	BP	GO:0060287	Epithelial cilium movement involved in determination of left/right asymmetry	Y	Y	
19	BP	GO:0030317	Flagellated sperm motility (a)	Y	Y	Modified by GOC/SYSCILIA project
20	BP	GO:0036159	Inner dynein arm assembly	Y	Y	
21	BP	GO:0035721	Intraciliary retrograde transport (a)	Y	Y	Modified by GOC/SYSCILIA project
22	BP	GO:0042073	Intraciliary transport (a)	Y	Y	Modified by GOC/SYSCILIA project
23	BP	GO:0044458	Motile cilium assembly	Y	Y	Added by GOC/SYSCILIA project
24	BP	GO:0036158	Outer dynein arm assembly	Y	Y	
25	BP	GO:0045724	Positive regulation of cilium assembly	Y	Y	Modified by GOC/SYSCILIA project
26	BP	GO:1901248	Positive regulation of lung ciliated cell differentiation	Y	Y	
27	BP	GO:0061512	Protein localization to cilium	Y	Y	Added by other
28	BP	GO:1902017	Regulation of cilium assembly	Y	Y	Added by GOC/SYSCILIA project
29	BP	GO:0003356	Regulation of cilium beat frequency	Y	Y	
30	BP	GO:0060296	Regulation of cilium beat frequency involved in ciliary motility	Y	Y	Modified by GOC/SYSCILIA project
31	BP	GO:0007288	Sperm axoneme assembly	Y	Y	Modified by GOC/SYSCILIA project
32	CC	GO:0005858	Axonemal dynein complex	Y	Y	Modified by GOC/SYSCILIA project
33	CC	GO:0005930	Axoneme	Y	Y	Modified by GOC/SYSCILIA project
34	CC	GO:0034464	BBSome	Y	Y	Modified by GOC/SYSCILIA project
35	CC	GO:0036064	Ciliary basal body (a)	Y	Y	Modified by GOC/SYSCILIA project
36	CC	GO:0060170	Ciliary membrane (a)	Y	Y	Modified by GOC/SYSCILIA project
37	CC	GO:0035253	Ciliary rootlet	Y	Y	Modified by GOC/SYSCILIA project
38	CC	GO:0035869	Ciliary transition zone	Y	Y	Modified by GOC/SYSCILIA project
39	CC	GO:0005929	Cilium	Y	Y	Modified by GOC/SYSCILIA project
40	CC	GO:0030991	Intraciliary transport particle A (a)	Y	Y	Modified by GOC/SYSCILIA project
41	CC	GO:0030992	Intraciliary transport particle B (a)	Y	Y	Modified by GOC/SYSCILIA project
42	CC	GO:0036038	MKS complex (a)	Y	Y	Modified by GOC/SYSCILIA project
43	CC	GO:0031514	Motile cilium	Y	Y	Modified by GOC/SYSCILIA project
44	CC	GO:0036157	Outer dynein arm	Y	Y	
45	CC	GO:0032391	Photoreceptor connecting cilium	Y	Y	Modified by GOC/SYSCILIA project
46	CC	GO:0001750	Photoreceptor outer segment	Y	Y	Modified by GOC/SYSCILIA project
47	CC	GO:0036126	Sperm flagellum	Y	Y	Modified by GOC/SYSCILIA project
48	CC	GO:0097225	Sperm midpiece	Y	Y	
49	BP	GO:1904158	Axonemal central apparatus assembly	N	Y	Added by GOC/SYSCILIA project
50	BP	GO:0032053	Ciliary basal body organization	N	Y	Modified by GOC/SYSCILIA project
51	BP	GO:0060294	Cilium movement involved in cell motility	N	Y	
52	BP	GO:0035720	Intraciliary anterograde transport	N	Y	Modified by GOC/SYSCILIA project
53	BP	GO:0035735	Intraciliary transport involved in cilium assembly	N	Y	Modified by GOC/SYSCILIA project
54	BP	GO:1903251	Multiciliated epithelial cell differentiation	N	Y	Added by other
55	BP	GO:1902018	Negative regulation of cilium assembly	N	Y	Added by GOC/SYSCILIA project
56	BP	GO:1902856	Negative regulation of non-motile cilium assembly	N	Y	Added by GOC/SYSCILIA project
57	BP	GO:1903568	Negative regulation of protein localization to ciliary membrane	N	Y	Added by GOC/SYSCILIA project
58	BP	GO:1903565	Negative regulation of protein localization to cilium	N	Y	Added by GOC/SYSCILIA project
59	BP	GO:1905515	Non-motile cilium assembly	N	Y	Added by GOC/SYSCILIA project
60	BP	GO:0003353	Positive regulation of cilium movement	N	Y	
61	BP	GO:1902857	Positive regulation of non-motile cilium assembly	N	Y	Added by GOC/SYSCILIA project
62	BP	GO:1903566	Positive regulation of protein localization to cilium	N	Y	Added by GOC/SYSCILIA project
63	BP	GO:1903441	Protein localization to ciliary membrane	N	Y	Added by GOC/SYSCILIA project
64	BP	GO:1904491	Protein localization to ciliary transition zone	N	Y	Added by other
65	BP	GO:0097499	Protein localization to non-motile cilium	N	Y	Added by GOC/SYSCILIA project
66	BP	GO:1903621	Protein localization to photoreceptor connecting cilium	N	Y	Added by other
67	BP	GO:1903546	Protein localization to photoreceptor outer segment	N	Y	Added by other
68	BP	GO:1903445	Protein transport from ciliary membrane to plasma membrane	N	Y	Added by GOC/SYSCILIA project
69	BP	GO:0097500	Receptor localization to non-motile cilium	N	Y	Added by GOC/SYSCILIA project
70	BP	GO:1902855	Regulation of non-motile cilium assembly	N	Y	Added by GOC/SYSCILIA project
71	CC	GO:0097729	9 + 2 motile cilium	N	Y	Added by GOC/SYSCILIA project
72	CC	GO:0097541	Axonemal basal plate	N	Y	Added by GOC/SYSCILIA project
73	CC	GO:1990716	Axonemal central apparatus	N	Y	Added by GOC/SYSCILIA project
74	CC	GO:1990718	Axonemal central pair projection	N	Y	Added by GOC/SYSCILIA project
75	CC	GO:0005879	Axonemal microtubule	N	Y	Modified by GOC/SYSCILIA project
76	CC	GO:0097546	Ciliary base	N	Y	Added by GOC/SYSCILIA project
77	CC	GO:0097543	Ciliary inversin compartment	N	Y	Added by GOC/SYSCILIA project
78	CC	GO:0097542	Ciliary tip	N	Y	Added by GOC/SYSCILIA project
79	CC	GO:0097539	Ciliary transition fiber	N	Y	Added by GOC/SYSCILIA project
80	CC	GO:0036156	Inner dynein arm	N	Y	
81	CC	GO:0030990	Intraciliary transport particle	N	Y	Modified by GOC/SYSCILIA project
82	CC	GO:1902636	Kinociliary basal body	N	Y	Added by GOC/SYSCILIA project
83	CC	GO:0060091	Kinocilium	N	Y	Modified by GOC/SYSCILIA project
84	CC	GO:0097730	Non-motile cilium	N	Y	Added by GOC/SYSCILIA project
85	CC	GO:0001520	Outer dense fiber	N	Y	Modified by GOC/SYSCILIA project
86	CC	GO:1990075	Periciliary membrane compartment	N	Y	Added by GOC/SYSCILIA project
87	CC	GO:0097227	Sperm annulus	N	Y	
88	CC	GO:0035686	Sperm fibrous sheath	N	Y	Modified by GOC/SYSCILIA project
89	CC	GO:0097228	Sperm principal piece	N	Y	

This table shows the ciliary GO terms used for annotation of mouse genes by experimental evidence codes in July 2013 and the additional GO terms used in December 2016. Of the 48 ciliary GO terms used in 2013, eight were no longer used in 2016. Seven of these were considered to be redundant with other existing terms and were thus merged into other ciliary GO terms; the eighth was too general for use in annotations of mouse genes. As of December 2016, an additional 41 ciliary terms, many of them newly added, have been used in experimental annotations of mouse genes for a total of 81 ciliary terms. (a) Term name changed since July 2013

**Table 4:** Initial ciliary curation status and prioritization (July 2013)

Ciliary curation status (experimental—July 2013)	# of genes			# genes by curation status
	Ciliary references	Other references	No references	
Genes with no ciliary annotations	30**	73**	83	186
Genes with BP only	9*	5*	11	25
Genes with CC only	24*	14*	15	53
Genes with both BP and CC	15	7	24	46
# genes by reference availability	78	99	133	310

Based on the existence of experimental annotations to ciliary BP and/or CC GO terms for each of the 310 mouse genes (see Table 3 for list of ciliary GO terms), we assigned each gene a ciliary curation status. Combined with the availability of literature, some of which was focused on cilia, we placed genes associated with relevant uncurated literature but with no ciliary annotations ** into the high priority category, while those with only a few ciliary annotations * were placed into the medium priority category

### Availability of ciliary literature for curation

We estimated the availability of uncurated relevant literature for this set of ciliary genes. As of 7/25/2013, these 310 genes were linked to over 7100 publications, not all of which were selected for GO curation, in the Mouse Genome Database. More than 1200 of these papers had already been curated for GO annotations. To obtain a minimum estimate of the number of publications with information relevant to cilia, the article titles of the remaining 5800 papers were searched for keywords (see Table 5), including some form of cilia, flagella, ciliogenesis, ciliopathy, or the name of a ciliopathic syndrome [31]. This identified 189 ciliary references linked to 97 mouse genes, many of which did not have annotations to any of the ciliary terms in BP or CC. While this method of identifying ciliary literature will have missed some ciliary papers, it was sufficient for our purpose to discover if papers focused on ciliary biology existed within the uncurated corpus for these genes, and allowed us to prioritize our attention on genes known to have relevant uncurated literature in the MGD system. For example, of the 186 genes lacking experimental annotations to ciliary GO terms, 83 were not associated with any uncurated papers and thus could not be further curated at the initial time point of the project. The remaining 103 genes without any ciliary annotations did have associated references, and for 30 of these genes we identified cilia-focused literature. Combining the curation status with respect to ciliary GO annotations with the availability of relevant literature, we assigned each gene to a priority curation class that focused curation towards genes lacking any experimental ciliary annotations and having available uncurated references (see Table 4 and Fig. 1).

**Table 5:** Keywords for preliminary identification of ciliary literature

Ciliary words	Disease syndrome words
Cilia/cilium	Alstrom
Flagella/flagellum	Bardet–Biedl
Ciliogenesis	BBS
Ciliopathy/ciliopathies	Joubert
	Leber
	McKusick
	Meckel
	MKS
	Nephronophthisis
	NPHP
	PKD
	polycystic kidney
	Senior-Loken

To identify references focused on cilia or ciliopathies among the set of papers linked to the mouse genes in our set, we used various forms of the words cilia, flagella, ciliogenesis, and ciliopathy, as well as the names of several ciliopathies [31] to search within the titles of the over 5800 papers linked to this set of mouse genes and not yet curated for GO annotations

### Annotation progress

Using the curation priority categories determined by the presence or absence of existing experimental annotation to ciliary terms, as well as availability of relevant publications, we then annotated the mouse genes corresponding to the SYSCILIA Gold Standard (SCGS) human gene list. In our workflow, when a given paper was curated, all annotations supported by the paper were made, not just those for genes on the ciliary list, following standard GO annotation practice at Mouse Genome Database (MGD) [57]. Thus, we also generated annotations for genes on the mouse cilia list that were not directly targeted for curation, as well as for additional genes not on this cilia list, using all relevant GO terms, not just ciliary GO terms. In addition, when curating papers that contained experiments on human or rat genes orthologous genes to those of the mouse, annotations would be made for the mouse genes with sequence orthology (ISO) evidence and the corresponding experimental annotations for human or rat would be generated when the mouse annotation data were loaded into UniProt via established GOC annotation procedures [58].

As of December 2016, 134 of the 310 mouse genes have been fully curated based on the available experimental literature for mouse. We have curated all available literature for most genes that are marked as “complete.” However, for some genes with a lot of published literature, e.g., *Ift88*, which had over 100 associated publications, we have scanned the abstracts of the available papers flagged for GO curation in the Mouse Genome Database and curated a selected subset of papers that results in the generation of a set of GO annotations representative of the known functions for the gene. A gene may have also been marked as “completed” for this curation project if we examined it and no literature was available—a category of genes that is regularly monitored for new literature.

### Improvements in representation of cilia within GO

During this project, we collaborated with John Van Dam of the SYSCILIA Consortium, and with the Gene Ontology Consortium ontology development team, to improve the representation of cilia-associated processes within the Gene Ontology vocabularies [8]. The focus on cilia provided by this collaborative effort more than doubled the number of cilia terms in the GO. From 85 cilia-related terms present before the project started in early 2013, we now count 180 terms (including 27 terms specific to giardia, dinoflagellates, or other protists) as of December 2016—more than double the original number of ciliary terms.

The number of cilia-related GO terms that are used in annotations for this set of mouse genes also expanded dramatically from 48 to 81 (see Table 3). Of these 81 ciliary GO terms used in 2016, 32 are new GO terms, and 27 of these new terms were added to GO by our collaboration with the SYSCILIA project to improve the representation of cilia biology within GO. This collaborative project also improved the definition or position within the ontology of 35 previously existing ciliary terms that have been used in annotation of mouse genes. Thus, 62 of 81 (about 75%) of the terms used for GO annotations of mouse genes in December 2016 were added or improved by this collaboration between the SYSCILIA and GO consortia. The combination of the addition of new terms and improvement of existing terms has greatly improved the representation of cilia within the Gene Ontology, and thus the ability to accurately annotate the functions of genes.

### Increases in GO annotations

To evaluate progress to date, we compared the original GO annotations for all 310 genes as of 7/25/2013 with the annotations present for the same set of genes on 12/24/2016. For these 310 genes, there has been a dramatic increase of over 1700 experimental annotations (see Table 2C). Over 450 of these annotations were to ciliary GO terms. For the 134 genes we have completed as of December 2016, the number of annotations to ciliary GO terms increased more than threefold (see Table 2D). While these data include annotations from all sources, MGI as well as other annotation groups, the majority of mouse annotations are generated by MGI. In addition, the dramatic increase in annotations for the genes we have “completed” compared to the ones we have not yet targeted suggests that our focused effort is responsible for much of this increase.

In addition to the increase in annotations to ciliary GO terms, there were over 400 new annotations to GO terms that, while not exclusively ciliary, are in areas that we observe frequently when annotating ciliary genes. For example, when annotating ciliary genes based on knockouts in mice, we frequently see defects in left/right patterning due to the requirement for functional nodal cilia as part of generating initial left/right asymmetry. It is also common to see defects in dorsal/ventral patterning due to the fact that Smoothened (*Smo*) signaling is regulated by changes in location to and from the cilium. The planar cell polarity pathway is of critical importance in the development of multiciliated epithelia. These early developmental events have downstream consequences in development of organs such as the brain, lungs, digestive system, kidneys, eyes, ears, and nose. Thus of the GO terms used in December 2016, we hand-selected 295 “related” GO terms (primarily from BP, but also 20 from CC and 10 from MF) that we feel are often relevant, though not necessarily exclusive, to ciliary biology (see Additional file 7) and an increase from the 253 “related” terms used in July 2013 (see Additional file 8). As well as the annotations to “ciliary” or “related” GO terms, 850 annotations were made to some of the nearly 1800 other GO terms now used in annotation of this list of cilia genes in mouse. For all three of these categories of GO terms, the fold increase in experimental annotations is significantly greater for “completed” genes compared to genes not yet targeted.

The number of annotations based on sequence similarity methods also increased dramatically, with over 1000 new sequence similarity annotations, over 300 of which are to ciliary or related GO terms. Some of this increase is due to the ISO annotations for mouse genes based on experimental work on human or rat that we made in the course of annotating papers characterizing genes from these other species as well as mouse. Some of the others are due to annotations for human genes made by the SYSCILIA Consortium [8] and propagated to sequence similarity annotations for the corresponding mouse genes [58].

At the beginning of the project, there were a number of annotations to the root term of a given aspect of the Gene Ontology indicating that the literature for the gene has been examined and there was nothing known at that time. These cases are tracked for emerging literature. During this annotation project, with the focused literature capture, there has been a large decrease in these cases, almost a 50% decrease for genes that have been targeted already, and about a 40% decrease for genes not yet targeted for curation.

We took advantage of the improved experimental annotations of mouse genes to make phylogenetic annotations. Based on our detailed experimental annotations for mouse BBsome and IFT subunits, we were able to generate phylogenetic annotations for twenty PANTHER families containing these genes via the PAINT methodology [40]. As the mouse experimental annotations were often the source evidence for phylogenetic propagation of annotations, this produced only a modest increase in phylogenetic annotations for mouse. However, the annotations of these genes in numerous other species, including human and rat, has been improved. These phylogenetic annotations targeted specifically for ciliary genes are only a portion of the new phylogenetic annotations generated for these 310 genes. Many were generated by other members of the PAINT team during on-going curation processes. However, all of them help improve our understanding of the functions of these genes.

### Assessing “curation status” and improvements in annotations per gene

In our initial evaluation of the “curation status” of these ciliary genes, we determined whether genes had annotations to ciliary BP terms, ciliary CC terms, both types of ciliary terms, or neither. We recognize that some genes may not be annotated to both BP and CC ciliary terms even when fully annotated, e.g., a regulator of cilium formation that is never localized to the cilium itself. Nevertheless, evaluating whether genes are annotated to ciliary terms in one or both of these GO aspects provides some indication of the status of ciliary annotation based on currently available literature.

To determine how many of these 310 genes had their ciliary “curation status” improved during this time period, we repeated our analysis using the experimental annotations for this set of genes as present in MGD’s MouseMine tool on 12/24/2016. Out of 1733 new experimental annotations to any GO term without a NOT qualifier (Table 2C), 472 (351 for 134 “completed” genes and 121 for 176 genes not yet targeted for curation) were to the expanded list of 81 ciliary GO terms (Table 3) used in annotations in December 2016 (Table 2B). Of the 134 genes completed, 57 had an improvement in ciliary curation status as did an additional 21 genes not yet targeted for curation (Table 6). In total, the number of these genes that are annotated with both BP and CC ciliary terms nearly doubled (from 42 to 78). Of these 78 genes with improved ciliary annotation status, 59 genes had lacked any ciliary annotations at the commencement of our annotation project. An additional 63 genes had increases in annotations to ciliary terms in one or both aspects of GO. Thus, nearly half (141) of the 310 mouse genes had an increase in the number of experimental GO annotations to ciliary GO terms during this time frame.

**Table 6:** Changes in experimental ciliary annotation #’s and curation status (July 2013–Dec 2016)

Changes in ciliary annotation #’s and curation status (July 2013–Dec 2016)	# genes completed	# genes not yet targeted	Total # genes
Increases in annotations resulting in improved curation status			
Only CC ciliary annotations ⇒ Both BP and CC ciliary annotations	10	4	14
Only BP ciliary annotations ⇒ Both BP and CC ciliary annotations	3	2	5
No ciliary annotations ⇒ Both BP and CC ciliary annotations	16	2	18
No ciliary annotations ⇒ BP ciliary annotations	9	6	15
No ciliary annotations ⇒ CC ciliary annotations	19	7	26
Total # genes with improved curation status	57	21	78
Changes in ciliary annotations but without curation status change			
Increase in both ciliary BP and CC terms	12	4	16
Increase in ciliary BP terms	1	3	4
Increase in ciliary CC terms	15	27	42
Decrease in ciliary CC terms		1	1
Total # genes with new annotations but unchanged curation status	28	35	63
Decrease in annotations resulting in decreased curation status			
Both BP and CC ciliary annotations ⇒ Only BP ciliary annotations	1	0	
Total # genes with decreased curation status	1	0	1
No change in ciliary annotations (genes still lacking ciliary annotations)	48	120	168
Total # genes	134	176	310

Comparing the annotations present in March 2016 to those present before the commencement of the annotation project, we have determined how many genes had an increase (or decrease, in the case of a single gene with a decreased annotation status due to removal of a single incorrect CC annotation) in the numbers of annotations to ciliary biological process (BP) or cellular component (CC) GO terms and how many of these genes also had an improvement in their “curation status” indicating new annotations in a GO aspect (either BP or CC) in which they previously did not have any ciliary annotations

### Assessing enrichment analysis

To focus specifically on the effect of our manual annotation project, we performed two enrichment analyses where we limited the annotations considered to those with experimental evidence codes and the query set was composed of the 134 genes we have “completed” at some point in time between July 2013 and December 2016. The VLAD tool at MGI [54, 55] provides a great deal of control to the user, providing options to upload the specific annotation file and ontology file desired, as well the ability to select which evidence codes should be considered for the analysis. VLAD also generates graphical visualizations of the most enriched terms as well as text files of the results available for download. The “before” analysis utilized the annotation and ontology data as of July 2013, while the “after” analysis used the data as of December 2016.

**Fig. 2:**
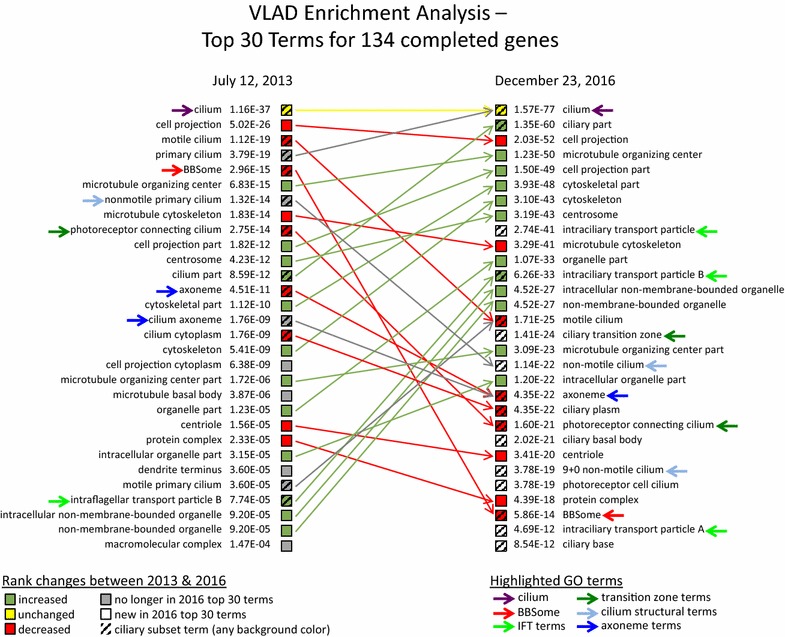
Comparison of top 25 most significant terms with experimental evidence. Enrichment analyses were performed using the VLAD web tool [54, 55] using mouse annotation and Gene Ontology files from either July 2013 or December 2016. The Query set of genes was composed of the 134 “completed” genes that were comprehensively annotated during this project. Only annotations with experimental evidence codes (IDA, IGI, IMP, IPI, IEP, EXP; the latter two of which are not present in the July 3013 GAF) were considered in the enrichment analysis; see Additional file 4 for acronym expansions of the Gene Ontology evidence codes [67]. Green squares: GO terms that rank higher using the current version of GO; yellow square: term that did not change in rank; red squares: terms that rank lower; gray squares: terms that have dropped out of the top 30 ranked results; white squares: terms that are among the top 30 when using the current annotations and version of GO, but not the July 2013 data; striped squares: ciliary subset terms (Table 3 lists terms that are used directly for experimental annotations of mouse genes. For the complete list of ciliary subset terms, including terms such as ‘ciliary part’ that are not used for direct annotation and other terms that are not used in annotation of mouse genes, see Additional file 3 from Roncaglia et al. [8]). GO terms of interest discussed in the text are highlighted with colored arrows as indicated on the key

Comparing the 30 most significant cellular component terms from each of these two analyses (Fig. 2 and Additional files 5 and 6), some differences are due largely to improvements in the ontology structure. As discussed in Rongaglia et al. [8], ‘cilium axoneme’ was merged into ‘axoneme’ since these two terms represented the same thing (dark blue arrows). In addition, terms such has ‘non-motile primary cilium’ were merged into more general terms such as ‘motile cilium’ and new terms were added based on a structural classification of cilium types, such as ‘9 + 0 non-motile cilium’ (light blue arrows). In all of these ontology revisions, annotations were not lost, but transferred to an appropriate replacement term.

A term that appears in the top 30 terms of the December 2016 data, but not the July 2013 data, is ‘ciliary transition zone.’ One contributor to this increase in significance may be the increase in annotations directly to the term ‘ciliary transition zone.’ However, an even larger factor is likely the fact that the ontology structure now recognizes that the ‘photoreceptor connecting cilium’ is a specialized type of ‘ciliary transition zone,’ contributing to the significance of the term ‘ciliary transition zone’ (dark green arrows). Interestingly, the number of genes with experimental annotations directly to ‘photoreceptor connecting cilium,’ which was already in the top 30 terms in July 2013, nearly doubled during the course of this annotation project (see Additional file 9).

There are also places where improvements in the experimental annotations are the main, or even sole, contributor to differences in the terms that show up in the top 30 most enriched terms, notably the addition of the terms ‘intraciliary transport particle’ and ‘intraciliary transport particle A’ into the top 30, as well as the dramatic increase in the rank of the term ‘intraciliary transport particle B’ (light green arrows) in the December 2016 data. In contrast, the term ‘BBSome’ (red arrow) is near the top of the most enriched terms in the July 2013 analysis, but has dropped in rank in the December 2016. Interestingly, the genes of the BBSome (being one of the areas that brought the recent research focus on the cilium to our attention) were well annotated prior to the beginning of this project. Thus, while the number of experimental annotations to this term in the set of 134 completed genes (see Table 7) did increase during this project, the number of genes annotated did not change and the term BBSome was surpassed by terms that increased in significance, dropping it to near the bottom of the top 30 most significant terms. In contrast, neither the A nor B subcomplexes of the IFT complex were well annotated before the start of this project with only two genes annotated to the term “intraciliary transport particle B.” However, as part of this project, both the IFT A (6 new annotations for 6 genes) and the IFT B subcomplexes [57 new annotations for 18 genes within the set of “completed” genes, as well as annotations to two other genes, Kif17 (present in SysCilia gene set but not “completed”) and Rabl2 (not present in SysCilia gene set)] have now been thoroughly annotated (see Table 7 and Additional files 5 and 6). Thus, with 18 genes in the query set out of a total of 20 genes annotated to this term, the term for the IFT B complex is one of the most significantly enriched terms on its own, with the general term for the IFT complex being even more significant as it incorporates the IFT A annotations as well.

**Table 7:** Increases in experimental and sequence based annotations to BBSome and IFT terms for completed genes

Gene symbol (IDs)		# annotations (July 2013)		# annotations (Dec 2016)	
		IDA	ISO	IDA	ISO
A. BBSome (GO:0034464)					
Bbs1	(MGI:1277215, Q3V3N7)	2	1	3	1
Bbs2	(MGI:2135267, Q9CWF6)	2	2	4	2
Bbs4	(MGI:2143311, Q8C1Z7)	2	1	3	1
Bbs5	(MGI:1919819, Q9CZQ9)	2	1	4	1
Bbs7	(MGI:1918742, Q8K2G4)	2	1	4	1
Bbs9	(MGI:2442833, Q811G0)	1	1	3	1
Ttc8	(MGI:1923510, Q8VD72)	2	1	4	1
Total		13	8	25	8
B. intraciliary transport particle A (GO:0030991)					
Ift122	(MGI:1932386, Q6NWV3)		1	1	1
Ift140	(MGI:2146906, E9PY46)		1	1	1
Ift43	(MGI:1923661, Q9DA69)		1	1	1
Ttc21b	(MGI:1920918, Q0HA38)		1	1	1
Wdr19	(MGI:2443231, Q3UGF1)		1	1	2
Wdr35	(MGI:1921932, Q8BND3)		1	1	1
Total			6	6	7
C. intraciliary transport particle B (GO:0030992)					
Cluap1	(MGI:1924029, Q8R3P7)			1	
Hspb11	(MGI:1920188, Q9D6H2)	1		2	
Ift172	(MGI:2682064, Q6VH22)			3	
Ift20	(MGI:1915585, Q61025)	1		5	
Ift22	(MGI:1914536, Q9DAI2)			3	
Ift27	(MGI:1914292, Q9D0P8)			4	1
Ift46	(MGI:1923818, Q9DB07)			4	
Ift52	(MGI:2387217, Q62559)			5	
Ift57	(MGI:1921166, Q8BXG3)			5	
Ift74	(MGI:1914944, Q8BKE9)			4	
Ift80	(MGI:1915509, Q8K057)			4	
Ift81	(MGI:1098597, O35594)			5	
Ift88	(MGI:98715, Q61371)			5	
Traf3ip1	(MGI:1921269, Q149C2)			3	
Ttc26	(MGI:2444853, Q8BS45)			2	
Ttc30a1	(MGI:1926052, Q99J38)			2	
Ttc30a2	(MGI:3700200, A2AKQ8)			1	
Ttc30b	(MGI:1919671, Q9CY00)			1	
Total		2		59	1

This table shows the number of experimental and sequence orthology annotations for genes in the set of 134 completed genes made directly to the BBSome (Panel A.), intraciliary transport particle A (Panel B.), and intraciliary transport particle B (Panel C.) terms in the cellular component aspect of GO before the start of this annotation project in July 2013 (from gene_association.mgi Revision 10039—*Jul 12 2013)* and as of December 2016. (from gene_association.mgi Revision 37787—Dec 23 2016). Both the MGI feature ID and the UniProt IDs are given

It is also notable that terms which were already significant in the July 2013 data, including the most enriched term ‘cilium’ (purple arrows) as well as the previously discussed term, ‘photoreceptor connecting cilium’ (dark green arrows), have much more significant *p* values in the December 2016 data, as was also observed during enrichment analysis of the entire human SCGS gene set in Rongaglia et al. [8]. It is hard to separate the effects of the dramatic ontology changes from the large increase in experimental annotations for these mouse genes, but it seems clear that both played a role in the effects we see in the enrichment analyses.

## Discussion

This annotation effort clearly made an improvement in the experimental annotation of mouse genes involved in ciliary biology. Using the GO phylogenetic workflow [40], we also propagated a subset of these experimental mouse annotations to many other species, including human. In addition, experimental annotations to mouse were propagated via 1:1 orthology relationships to numerous vertebrate species including rat and human, as part of a pipeline utilizing EnsemblCompara GeneTrees [59] run by the Gene Ontology Annotation (UniProt-GOA) project at the European Bioinformatics Institute (EBI) [39]. Thus, our focused effort on improving the GO annotations of mouse cilia genes also contributed to improve the annotation of many other species, including human. The work shown here and also that in Roncaglia et al. [8] demonstrate the impact the combination of our ontology work and annotation projects have had in improving the significance of ciliary GO terms in enrichment analyses of mammalian gene sets.

This work demonstrates a workflow that can be applied generally in any case where research on model organisms from vertebrates to yeast expands our efforts to understand a disease or health condition. Starting with a set of human genes of interest and progressing to the identification of the corresponding orthologs in a model organism provides a target set of genes for annotation in the model organism. Identification of which genes in the targeted set do not already have experimentally supported GO annotations in the biological area of interest can help determine which genes in the model organism are highest priority for curation. Determining which genes have available literature also increases the efficiency of the curation process. In this project, our evaluation of the available literature for each gene depended heavily on the literature triage pipeline at MGI to identify relevant literature, which was a great head start compared to searching PubMed directly. Inclusion of the abstracts could improve the ability of this step to identify relevant papers in future projects. Combining knowledge of which genes most needed annotation with which ones had available literature allowed curation to be efficiently directed at high-priority target genes.

Starting with the 310 mouse genes that correspond to the human SCGS set, our highest priorities were the 103 genes that lacked any experimental ciliary annotations and that had uncurated literature, whether explicitly about cilia or not. As of this analysis, we have completed 54 of these high-priority genes, slightly more than half. However, a majority of the genes we completed overall (80) were from categories we were not targeting, either genes that already had experimental ciliary annotations in one or both of BP and CC, or genes that lacked any associated uncurated references, regardless of ciliary annotation status (see Table 8). MGI’s general practice of making all annotations in a paper, not just those for the target gene that initiated curation of the paper, provides an explanation for some of the curation of genes in lower priority categories. In some cases, making annotations for secondary genes that co-occur in papers selected initially for other primary genes may result in “completion” of those secondary genes as all available literature associated with them has been curated.

**Table 8:** Percent completion of genes within initial curation priority categories

Type of experimental annotations and availability of references July 2013	Total # genes	# genes NOT “completed”	# genes “completed”	% “completed”	Curation notes for “complete” genes
No ciliary annotations, associated references—high priority					
No ciliary annotations—ciliary references [#1]	30	18	12	40.0%	6 of 12 (2 Bardet–Biedl, 2 dynein, 2 IFT)
No ciliary annotations—other references [#2]	73	31	42	57.5%	5 of 42 (3 dynein, 1 IFT, 1 sperm flagellum)
Ciliary annotations in either BP or CC—medium priority					
No ciliary BP annotations—ciliary references [#3]	24	18	6	25.0%	4 of 6 (1 BBSome, 3 IFT)
No ciliary CC annotations—ciliary references [#3]	9	6	3	33.3%	1 of 3 (1 CPLANE)
No ciliary BP annotations—other references [#4]	14	12	2	14.3%	
No ciliary CC annotations—other references [#4]	5	3	2	40.0%	1 of 2 (1 sperm flagellum)
No ciliary annotations in BP and/or CC, no references—low priority					
No ciliary annotations—no references [#5]	83	49	34	41.0%	17 of 34 (1 Bardet–Biedl, 1 CPLANE, 9 dynein, 6 IFT)
No ciliary CC annotations—no references [#6]	11	7	4	36.4%	1 of 4 (1 CPLANE)
No ciliary BP annotations—no references [#6]	15	6	9	60.0%	6 of 9 (2 Bardet–Biedl, 3 IFT, 1 sperm flagellum)
Ciliary annotations in both BP and CC—low priority					
Both BP and CC ciliary annotations—ciliary references [#7]	15	6	9	60.0%	6 of 9 (2 Bardet–Biedl, 2 IFT, 1 dynein, 1 sperm flagellum)
Both BP and CC ciliary annotations—other references [#7]	7	6	1	14.3%	1 of 1 (1 IFT)
Both BP and CC ciliary annotations—no references [#8]	24	14	10	41.7%	8 of 10 (2 BBSome, 6 IFT)
Totals	310	176	134	43.2%	

This table shows the various curation priority status categories and number of genes in each category, as well as the number of genes that were and were not completed at some point in time during the annotation project to date and % completed per category. The categories “high priority,” “medium priority,” and “low priority,” as well as the numbers in square brackets, e.g., [#1], correspond to prioritization categories on the prioritization flow chart (Fig. 1); note that some categories here have been grouped together on the flow chart. The curation notes for “completed” genes indicates the number of genes that were present in a biological area on which curation was focused as well as the total number of “completed” genes in each curation priority grouping. For each biological focus area, the number of genes is indicated. The biological focus areas included in the curation notes column are indicated for the relevant genes in the note column of Additional file 1

Scanning the list of genes completed (see Additional file 1) as sorted by completion date reveals several clusters of related genes completed within short time periods, e.g., genes for Bardet–Biedl syndrome, IFT, sperm flagellum, dyneins, and CPLANE. In these cases, there was generally a group of papers on a related set of genes. When this occurs, there is an increased efficiency to curate multiple related papers together as increased familiarity with the biology often improves the accuracy and detail of the annotations as well as the speed of curation. The intraflagellar transport particle is a particularly good example. Initially, a gene (likely Traf3ip1, aka Ift54) encoding a member of this complex was selected from the high-priority target list. Upon examination, it was apparent that there was a good deal of literature, most of which was not indexed to all members of the IFT complex mentioned within the paper, but perhaps only to a particularly significant one that was mentioned in the abstract. In addition, despite the fact that the majority of the IFT genes were annotated to at least one ciliary GO term, there was not much specificity in the existing experimental annotations, with only a handful of annotations to terms indicating their role in intraflagellar transport or their presence in the intraflagellar transport particle. Thus, examination of this initially prioritized gene revealed the need for curation of the entire IFT complex, resulting in several times where the completion dates indicate work on a group of IFT genes, eventually resulting in comprehensive experimental annotation of both the IFT A and IFT B complexes in mouse. It is also worth noting that while the human IFT A complex had already been annotated to the term ‘intraflagellar transport particle A’ experimentally, the IFT B complex had not. Even as of the December 2016 time point, these mouse annotations we generated are still the main source of detailed annotations for the human IFT B genes, based on their homology with the mouse genes.

In another case, once we had learned about the biology of human axonemal dynein genes as discussed in Roncaglia et al. [8], it was logical to see if it was possible to apply that knowledge to the annotation of mouse dyneins. Targeting the remaining mouse dynein genes for curation resulted in annotations for a couple of high-priority genes and also in examination of several genes from the category of genes without either ciliary annotations or associated references in MGI. This latter category of genes are lower priority solely due to lack of associated literature, but are actually highly desirable to annotate, as new publications often provide newly discovered information. In this case, there was still no literature associated with these genes in MGI and searching in PubMed did not reveal additional literature for mouse dyneins not already present in MGI, so very quickly we were able to mark several of these genes as “complete” as of that date.

There are some additional reasons we annotated genes outside of the high-priority category. In some cases, genes on this list showed up on other prioritization lists for MGI GO curation, such as one that alerts us to the presence of new papers for genes that have an annotation with the ‘ND’ evidence code indicating no knowledge as of the date of annotation, with especially high prioritization for genes associated with diseases cataloged by OMIM [60]. This was the case with a number of Bardet–Biedl syndrome genes.

Another very good reason to curate genes in lower priority categories was the receipt of a request for curation from a community member, such as one prompted by a conversation at a meeting, to curate genes present in or interacting with the CPLANE complex involved in establishment of planar cell polarity. This resulted in the curation of three genes (Fuz, Intu, and Wdpcp) in non-targeted categories on our list, as well as Jbts17, a gene not present in the SCGS-derived set and not previously characterized. Input from the community is incredibly valuable in helping us target our curation to genes where new information is available.

These examples illustrate some of the reasons we chose to curate genes outside of the 103 genes in the highest priority categories. However, they also demonstrate some potential opportunities for improvement. Our strategy for prioritizing genes utilized both the GO terms associated with experimental evidence and the associated references. There are issues worth considering with each of these.

Assessment of which GO terms should be considered for determining “initial annotation status” is worth further comment. As discussed earlier, the majority of the IFT genes were excluded from the priority set for annotation due to having at least one experimental annotation to any ciliary GO term, including the very general term ‘cilium.’ However, very few of these genes had experimental annotations with much specificity. None of the six members of the IFT A complex and only two of 18 members of the IFT B complex were annotated to the GO terms representing these complexes prior to our project. Thus in this case, inclusion of the most general ciliary GO terms like ‘cilium’ may have excluded too many genes from the high-priority set. Another consideration for future projects might be whether to consider genes that have annotations transferred by sequence similarity methods, particularly for 1:1 orthologs (ISO evidence), as already done. In the IFT example, this would have excluded all of the IFT A genes as the orthologous human genes were annotated to the specific term “intraflagellar transport particle A” with experimental evidence prior to our project. In contrast, none of the human IFT B genes were annotated to the term “intraflagellar transport particle B” (see Table 7). Possibly, the combined application of eliminating very general ciliary GO terms and inclusion of ISO annotations could possibly have indicated that IFT A genes were lower priority for further annotation than IFT B genes. While possibly more difficult than improving the literature triage and indexing processes, computational analysis of existing GO annotations to help prioritize which genes are annotated with very general terms versus specific leaf node terms in a given area could provide significant value.

## Conclusions

In summary, our prioritization strategy was effective at identifying genes in need of annotation in this area of biology. For future applications, refinements may be possible to both the identification of associated literature and to the granularity of the set of GO terms that constitute the target area of biology, as well as the categories of evidence considered, to define the initial annotation status, and thus priority for curation, of genes in the set. While this prioritization guides the choice of genes to select for annotation, it is also flexible to take advantage of opportunities to curate groups of related genes together, leading to increased efficiency for situations such as the IFT complex, or to additions to the set of ciliary genes that are detectable bioinformatically via GO functional annotations, such as the case *Jbts17*, where as of 1/10/2018 the human ortholog is still annotated largely based on the mouse annotations via “GO projections using Ensembl orthologs” [61]. Thus, this type of prioritization strategy may help maximize the effectiveness of limited curation resources.

Following our prioritization strategy for curation, we have improved the Gene Ontology annotations available, both with ciliary-related and other terms, for mouse genes considered orthologous to human genes in the SYSCILIA Consortium’s Gold Standard list and thereby also improved the GO annotations available for the human gene set. In addition, ciliary-related terms in the Gene Ontology itself were improved in collaboration with GO ontology developers [8], greatly increasing the specificity of the annotations we were able to make. This combination of improvements in the ontology as well as increases in the number and specificity of the annotations made dramatic improvements to the ability of enrichment analysis tools to identify when ciliary processes are significant.

Going forward, we will continue to focus on ciliary genes in the mouse for annotation. One continuing strategy will be to target the remaining mouse genes corresponding to the SYSCILIA Gold Standard list, prioritizing genes that are most in need of annotation. However, we are aware this list is only a beginning. There are many more cilia genes not on this list. In MGI, using MouseMine on 1/12/2018, there are currently 715 mouse genes annotated to a ciliary GO term [62] (see Additional file 10), 481 of which are not listed in the SCGS (311 of these 481 with experimental evidence, see Additional file 11 [63]). Even more strikingly, the Cildb V3.0 database [38], which contains data from numerous high-throughput ciliary studies from 15 different ciliated species, indicates that nearly 3500 mouse genes have high-throughput evidence from proteomic studies in mouse that suggests presence in cilia, centrioles, or related structures. When data from other species are considered via orthology relationships, Cildb lists nearly 8800 mouse genes with evidence for presence in some ciliary or related structure (see Additional file 12) [64]. However, despite the fact that this list of mouse genes with evidence for a ciliary or related localization in Cildb is much larger than the SCGS-derived list we focused on here, over a third (107 of 310) of the mouse SCGS genes do not have ciliary evidence performed in mouse and 42 of the mouse genes in the SCGS set do not have evidence from any species in Cildb (see Additional file 1). In the annotation work we describe here, we have not yet targeted 33 of these 42 mouse SCGS genes without any ciliary evidence in Cildb, suggesting that these genes might be particularly good targets going forward. Combining the data from Cildb within our workflow protocol might be a productive approach for identification of genes which would benefit from individual attention.

These improvements to the GO ciliary annotations for mouse genes, and to their corresponding human genes, are especially important to researchers studying ciliopathies, whether via model organisms such as the mouse [24, 36] or directly with human patients. Comprehensive annotation of ciliary genes will facilitate the identification by future studies of a role in cilia in a phenotype or disease where such a role may not have been previously known, such as the discovery that ciliary defects are a major contributor congenital heart disease [65]. In addition, as those performing large-scale mutant screens follow on to characterize individual candidate genes, such as *Jbts17* [66], our continuing work targeted on ciliary genes will generate GO functional annotations based on the experimental characterizations of these genes. In this way, our work will improve the GO knowledge representation of this exciting and rapidly developing area of biology, making it more accessible to genome wide expression studies, mutant screens, and other large scale or bioinformatic analyses.

## Additional files

**Additional file 1.** Curation of mouse homologs of the SYSCILIA Gold Standard human genes.

**Additional file 2.** Initial GO annotations as of 7/25/2013.

**Additional file 3.** Final GO annotations as of 12/24/2016.

**Additional file 4.** GO evidence codes.

**Additional file 5.** VLAD enrichment analysis with July 2013 annotations and ontology.

**Additional file 6.** VLAD enrichment analysis with December 2016 annotations and ontology.

**Additional file 7.** GO terms and classifications as of 12/24/2016.

**Additional file 8.** GO terms and classifications as of 7/25/2013.

**Additional file 9.** Increases in experimental annotations to ‘ciliary transition zone’ and ‘photoreceptor connecting cilium’ terms for “completed” genes.

**Additional file 10.** Annotations to ciliary GO terms in MGI as of 1/12/2018.

**Additional file 11.** All annotations in MGI to non-SysCilia Gold Standard genes having ciliary annotations on 1/12/2018.

**Additional file 12.** Mouse genes with at least 1 piece of experimental evidence in Cildb on 2/27/2018.

## Abbreviations

BP: biological process
CC: cellular component
EBI: European Bioinformatics Institute
GAF: gene association file
GO: Gene Ontology
GOC: Gene Ontology Consortium
MF: molecular function
PAINT: Phylogenetic Annotation and INference Tool
PANTHER: Protein ANalysis THrough Evolutionary Relationships
SCGS: SYSCILIA Gold Standard
SYSCILIA: a systems biology approach to dissect cilia function and its disruption in human genetic disease
UniProt-GOA: Gene Ontology Annotation Database at the Universal Protein Resource
VLAD: visual annotation display
