# Supplementary material for: Sensing the cilium, digital capture of ciliary data for comparative genomics investigations
Source: Cilia. 2018 Apr 19;7:3. doi: 10.1186/s13630-018-0057-0 (PMC5907423; doi:10.1186/s13630-018-0057-0)
Supplement: Supplementary file 4 — Additional file 4. GO evidence codes. [file 13630_2018_57_MOESM4_ESM.docx]

**GO Evidence Codes**

**Experimental Evidence codes**

EXP - Inferred from Experiment

IDA - Inferred from Direct Assay

IPI - Inferred from Physical Interaction

IMP - Inferred from Mutant Phenotype

IGI - Inferred from Genetic Interaction

IEP - Inferred from Expression Pattern

**Curatorial Statement codes**

**unknown** - annotation that function is unknown

ND - No biological Data available

**curator statement** - functional annotation based on curator biological knowledge

IC - Inferred by Curator

**Author Statement evidence codes**

TAS - Traceable Author Statement

NAS - Non-traceable Author Statement

**Computational Analysis evidence codes**

**sequence** - annotations based on sequence analysis, generally pairwise or small groups of sequences

ISS - Inferred from Sequence or structural Similarity

ISO - Inferred from Sequence Orthology

ISA - Inferred from Sequence Alignment

ISM - Inferred from Sequence Model

IGC - Inferred from Genomic Context

**phylogenetic** - annotations based on examination of sequences within phylogenetic trees

IBA - Inferred from Biological aspect of Ancestor

IBD - Inferred from Biological aspect of Descendant

IKR - Inferred from Key Residues

IRD - Inferred from Rapid Divergence

**other computational analysis** - typically combinations of multiple types of evidence/analysis

RCA - Inferred from Reviewed Computational Analysis

**Automatically-Assigned evidence code**

IEA - Inferred from Electronic Annotation
