# Supplementary material for: Sensing the cilium, digital capture of ciliary data for comparative genomics investigations
Source: Cilia. 2018 Apr 19;7:3. doi: 10.1186/s13630-018-0057-0 (PMC5907423; doi:10.1186/s13630-018-0057-0)
Supplement: Supplementary file 9 — Additional file 9. Increases in experimental annotations to ‘ciliary transition zone’ and ‘photoreceptor connecting cilium’ terms for “completed” genes. [file 13630_2018_57_MOESM9_ESM.docx]

**Increases in experimental annotations to ‘ciliary transition zone’ and ‘photoreceptor connecting cilium’ terms for “completed” genes**

| **Direct Experimental Annotations to GO term per Gene** | | **# July 2013** | **# in Dec 2016** |
| --- | --- | --- | --- |
| **ciliary transition zone (GO:0035869)** | | **2** | **6** |
| **Cc2d2a** | (MGI:1924487, Q8CFW7) | 1 | 2 |
| **Cep131** | (MGI:107440, Q62036) |  | 1 |
| **Mks1** | (MGI:3584243, Q5SW45) | 1 | 2 |
| **Ttbk2** | (MGI:2155779, Q3UVR3) |  | 1 |
| **photoreceptor connecting cilium (GO:0032391)** | | **11** | **21** |
| **1810043G02Rik** | (MGI:1915134, Q8C6G1) |  | 1 |
| **Adgrv1** | (MGI:1274784, Q8VHN7) |  | 1 |
| **Bbs4** | (MGI:2143311, Q8C1Z7) |  | 1 |
| **Cep290** | (MGI:2384917, Q6A078) | 2 | 3 |
| **Ift122** | (MGI:1932386, Q6NWV3) | 1 | 1 |
| **Ift140** | (MGI:2146906, E9PY46) | 1 | 2 |
| **Ift20** | (MGI:1915585, Q61025) |  | 1 |
| **Ift52** | (MGI:2387217, Q62559) | 1 | 1 |
| **Ift57** | (MGI:1921166, Q8BXG3) | 1 | 1 |
| **Ift88** | (MGI:98715, Q61371) | 2 | 2 |
| **Mak** | (MGI:96913, Q04859) |  | 1 |
| **Nphp1** | (MGI:1858233, Q9QY53) | 1 | 1 |
| **Nphp4** | (MGI:2384210, P59240) |  | 1 |
| **Ttc8** | (MGI:1923510, Q8VD72) | 1 | 1 |
| **Ush2a** | (MGI:1341292, Q2QI47) |  | 2 |
| **Wdr19** | (MGI:2443231, Q3UGF1) | 1 | 1 |
|  |  | **13** | **27** |
